# Metabolic output defines *Escherichia coli* as a health-promoting microbe against intestinal *Pseudomonas aeruginosa*

**DOI:** 10.1038/s41598-019-51058-3

**Published:** 2019-10-08

**Authors:** Theodoulakis Christofi, Stavria Panayidou, Irini Dieronitou, Christina Michael, Yiorgos Apidianakis

**Affiliations:** Department of Biological Sciences, University of Cyprus, Nicosia, Cyprus

**Keywords:** Bacterial genetics, Pathogens

## Abstract

Gut microbiota acts as a barrier against intestinal pathogens, but species-specific protection of the host from infection remains relatively unexplored. Although lactobacilli and bifidobacteria produce beneficial lactic and short-chain fatty acids in the mammalian gut, the significance of intestinal *Escherichia coli* producing these acids is debatable. Taking a Koch’s postulates approach in reverse, we define *Escherichia coli* as health-promoting for naturally colonizing the gut of healthy mice and protecting them against intestinal colonization and concomitant mortality by *Pseudomonas aeruginosa*. Reintroduction of faecal bacteria and *E*. *coli* in antibiotic-treated mice establishes a high titre of *E*. *coli* in the host intestine and increases defence against *P*. *aeruginosa* colonization and mortality. Strikingly, high sugar concentration favours *E*. *coli* fermentation to lactic and acetic acid and inhibits *P*. *aeruginosa* growth and virulence in aerobic cultures and in a model of aerobic metabolism in flies, while dietary vegetable fats - not carbohydrates or proteins - favour *E*. *coli* fermentation and protect the host in the anaerobic mouse gut. Thus *E*. *coli* metabolic output is an important indicator of resistance to infection. Our work may also suggest that the lack of antimicrobial bacterial metabolites in mammalian lungs and wounds allows *P*. *aeruginosa* to be a formidable microbe at these sites.

## Introduction

*Escherichia coli* and streptococci are the first bacteria to colonize the gastrointestinal tract of humans upon birth, paving the way for the establishment of species of the *Bifidobacterium*, *Bacteroides* and other genera^1^. *Bifidobacterium* and *Lactobacillus* strains are considered efficient fermenters in the human gut^2,3^. *E*. *coli* on the other hand thrives aerobically, but may also ferment carbon sources anaerobically to produce short-chain fatty acids (SCFAs) such as acetic acid and related metabolic products such as lactic acid^4,5^. While it is an effective colonizer of the healthy anaerobic mammalian gut, commensal *E*. *coli* also has a fitness advantage upon gut inflammation and concomitant host-derived nitrate production^5^. Interestingly, the probiotic *E*. *coli* strain Nissle 1917 (EcN) is particularly beneficial to ulcerative colitis patients in maintaining disease remission^6–8^. EcN induces host immune defence against pathogens^9,10^, strengthens the intestinal barrier^11,12^, and directly inhibits pathogenic *E*. *coli* strains^13,14^. Yet the beneficial role of *E*. *coli* has so far only been demonstrated for EcN and is not linked to lactic acid and SCFA production, while lactic acid bacteria, such as lactobacilli and bifidobacteria, are considered the main probiotic fermenters in the mammalian gut.

Antibiotics can greatly reduce microbiota diversity and promote dysbiosis early in life^15^. In children and adults, opportunistic pathogens can take advantage of the antibiotic effect on commensal bacteria to infect the gut^16^. One such pathogen is the gram-negative human opportunistic bacterium *Pseudomonas aeruginosa*, which is frequently found in hospital-acquired infections^17^. While not a common clinical problem in the gut, *P*. *aeruginosa* colonizes the gastrointestinal tract of many hospitalized patients and to a lesser extent of healthy individuals^18–21^. *P*. *aeruginosa* can nevertheless cause frequent and severe wound and lung infections in immunocompromised individuals and the ears and eyes of seemingly healthy people^22^. It is responsible for more than 50,000 infections per year in the U.S. alone, causing acute, chronic and relapsing infections due to a wide variety of virulence factors. Many of its virulence genes are controlled by quorum sensing (QS), a bacterial communication system that promotes synchronized microbial behaviours such as the production of the oxidative agent pyocyanin by *P*. *aeruginosa*^23^.

Here we interrogate the contribution of *E*. *coli* in controlling *P*. *aeruginosa* intestinal colonization in a nutrient-dependent manner. We apply the Koch’s postulates in reverse to prove a causal role of commensal *E*. *coli* in fending off *P*. *aeruginosa* infection. We found that: (**a**) *E*. *coli* is detectable through culture-independent methods (16S sequencing) in the faeces of untreated mice but not of antibiotic-treated mice, which become susceptible to infection; (**b**) A candidate health-promoting commensal *E*. *coli* strain was isolated through culture-dependent microbiological analysis and archived as a pure culture in the laboratory; (**c**) This mouse *E*. *coli* strain and other *E*. *coli* strains ameliorate *P*. *aeruginosa* infection when introduced into antibiotic-treated mice; (**d**) The administered health-promoting *E*. *coli* strains can be identified in high titres in the faeces of mice in which resistance to infection was improved. Moreover, assessing three extremes and a conventional diet in mice we find that, while sugars are fermented by various *E*. *coli* strains to lactic and acetic acid in culture and in flies aerobically, in the anaerobic mouse gut a vegetable-fat-based rather than a carbohydrate- or protein-based diet boosts lactic acid production and helps *E*. *coli* to inhibit *P*. *aeruginosa*. Our findings support the notion that unbalanced diets or the use of antibiotics may eliminate not only lactic acid bacteria but also commensal *E*. *coli*, imposing a gut environment conducive to *P*. *aeruginosa* infection due to the depletion of lactic acid and SCFAs.

## Methods

### Bacterial strains

*Pseudomonas aeruginosa* strain UCCBP 14 (PA14) and isogenic gene deletion mutants *Δmvfr*, *ΔphzS*, *ΔphzS* and *ΔrhlR/ΔlasR* were previously described^24,25^. *E*. *coli* MGH is a human isolate obtained from Prof. Elizabeth Hohmann at Mass General Hospital (Boston, USA). Mouse *E*. *coli* (*E*. *coli* CD1) was isolated from the faeces of CD1 mice for this study and validated through colony PCR and biochemical analysis i.e. being positive for indole production and growth on selective chromogenic Tryptone Bile X-glucuronide (TBX) agar plates. Laboratory *E*. *coli* BW25113 and KEIO collection strains, including *Δpgi*, *ΔadhE*, *ΔatpC*, *Δpta* and *ΔldhA*, were previously described^26^. Laboratory *E*. *coli* BW25113 and *Δtna*, *ΔsdiA*, *ΔluxS*, strains were previously described^27^. Enteropathogenic (EPEC) *E*. *coli* O127:H6 E2348/69 was obtained from Prof. Tassos Economou and was previously described^28^.

### Bacteria handling for in-culture experiments

*E*. *coli* and *P*. *aeruginosa* strains were grown at 37 °C overnight with shaking at 200 rpm in liquid LB from frozen LB-20% glycerol stocks. Cultures were then diluted to OD_600nm_ 0.01 in fresh sterile LB to establish mono- or co-cultures. Sucrose or glucose was added to a final concentration of 4% w/v during growth assessments. Bacterial supernatants were produced by overnight bacterial cultures filter-sterilized and mixed in 1:1 volume ratio with fresh LB broth. Selective plates contained 50 μg/ml rifampicin for *P*. *aeruginosa* and 60 μg/ml kanamycin for *E*. *coli* Keio collection or TBX agar for wild-type *E*. *coli*.

### Fly survival

For aerobic growth, strains were grown at 37 °C overnight with shaking at 200 rpm in liquid LB from frozen LB-20% glycerol stocks and then diluted to OD_600nm_ 0.01 in fresh sterile LB grown over day to OD_600nm_ 3. For anaerobic growth, strains were grown at 37 °C for 72 hours without shaking in liquid BHI from frozen BHI-20% glycerol stocks to OD_600nm_ 1–2. Cultures were then pelleted and diluted to a final OD_600nm_ 0.15 per strain in a 4% sugar (sucrose or glucose), 10% sterile LB infection medium. Wild-type Oregon R *Drosophila melanogaster* female flies 3–5 days old were starved for 6 hours prior to infection. 5 ml infection medium was added on a cotton ball at the bottom of a fly vial. Each vial contained 10 to 15 flies and observed twice a day for fly survival^29^.

### Fly colonization

Germ-free flies were generated through dechorionation of collected eggs in 50% bleach. Adult Oregon R 3–5-day-old female flies were infected for 24 hours with a single bacterial culture or a mix of cultures grown as mentioned above, pelleted and diluted to a final OD_600nm_ 0.02 per strain in a 4% sugar (sucrose or glucose) medium. Flies were then transferred to modified falcon tubes and maintained there with 200 μl 2% or 4% of sucrose or glucose as previously described^24^. At day 2 and day 5 flies were homogenized using the Qiagen Tissuelyser LT for 5 minutes at 50 Hz. Bacteria CFUs were enumerated on selective plates after overnight incubation at 37 °C.

### KEIO *E.coli* gene deletion library screen

The Keio *E*. *coli* collection of gene knockouts was acquired from the Japanese National Institute of Genetics and contains 3884 *E*. *coli* mutants with unique gene deletions. Strains were grown overnight in sterile 96-well clear flat bottom plates containing 200 μl of sterile LB broth at 37 °C and 200 rpm shaking. *P*. *aeruginosa* was grown in glass tubes at standard overnight conditions. Over day co-cultures were incubated at 37 °C and 200 rpm in 96-well plates starting with 1:100 dilutions of *P*. *aeruginosa* and *E*. *coli* mutant overnight cultures in 200 μl LB broth supplemented with 4% glucose. At 24 hours pyocyanin production was observed visually using as positive controls PA14 monocultures and co-cultures of PA14 with *E*. *coli* mutants lacking inhibitory properties (e.g. *Δpgi*). Bacterial growth was measured at OD_600nm_ on a plate reader. Bacterial co-cultures typically exhibit half the optical density of PA14 monocultures. Thus co-cultures with optical density equal to or higher than PA14 monocultures indicated antagonistic interactions.

### Animal diets

*Drosophila melanogaster* Oregon R flies were reared in a cornmeal, yeast and sugar diet at 25 °C in a 12-hour day and night cycle. CD1 mice were reared 5–6 individuals per cage at 24 °C in a 12-hour day and night cycle. Standard chow diet was obtained from Mucedola s.r.l Italy (#4RF25 a complete balanced diet containing mainly starch 35.18%, sucrose 5.66%, crude protein 22%, crude oil 3.5%). Specialized diets based on either vegetable fats, carbohydrates or protein were manufactured by Mucedola s.r.l (#PF4550, PF4551 and PF4552) per Table 1 below^30^.

**Table 1:** Composition of macronutrient diets (% by weight).

	Carbohydrate	Fat	Protein
Corn starch	58.11	0.00	0.00
Powdered sugar	29.06	0.00	0.00
Casein	0.00	0.00	87.17
dl-Methionine	0.11	0.20	0.11
Vegetable shortening*	0.00	75.12	0.00
AIN-76A vitamin mix**	0.77	1.49	0.77
AIN-76A mineral mix**	3.07	5.95	3.07
Choline chloride	0.18	0.34	0.18
Cellulose (Alphacel)	8.72	16.91	8.72
Energy density, kcal/g	3.53	6.85	3.53

*Crisco brand, a blend of soybean oil, fully hydrogenated palm oil, and partially hydrogenated palm and soybean oils. Contains 50% polyunsaturated fat, 20.8% monounsaturated fat, 0% trans fat and 25% saturated fat per weight.

**Vitamin (A and D3) and mineral (Fe, Mn, Zn, Cu, I, Se) mixes contain 97% and 12% sucrose, respectively.

### Ethics statement

Animal protocols were approved by the Cyprus Veterinary Service inspectors under the license number CY/EXP/PR.L6/2018 for the Laboratory of Prof. Apidianakis at the University of Cyprus. The veterinary services act under the auspices of the Ministry of Agriculture in Cyprus and the project number is CY.EXP101. These national services abide by the National Law for Animal Welfare of 1994 and 2013 and the Law for Experiments with Animals of 2013 and 2017. All experiments were performed in accordance with these guidelines and regulations.

### Mouse colonization assay

Female CD1 mice 7–8 weeks old were treated with an antibiotic cocktail of 0.1 mg/ml Rifampicin, 0.3 mg/ml Ampicillin and 2 mg/ml Streptomycin for 6 days to reduce endogenous gut bacteria. Subsequently, PA14 was provided daily for 7 days in the drinking water prepared from an over-day culture of OD_600nm_ 3, centrifuged at 4610 RCF for 5 minutes to collect bacteria and diluted 1:10 to obtain ~3 × 10^8^ bacteria/ml. Following infection (Day 0 of PA14 colonization) *E*. *coli* was provided for 1 day at the same concentration and CFUs for both bacteria were measured every other day from homogenized and plated mouse faeces.

### 16S Metagenomic

Mouse faecal samples were collected in Eppendorf tubes, weighed, snap frozen and stored at −80 °C. Bacterial DNA was extracted using the QIAamp DNA Stool Mini Kit (Qiagen). 16S Sequencing was performed using the Illumina metagenomics analyser. Kraken software was used to assign taxonomic sequence classification.

### Mouse survival assay

Female CD1 mice 7–8 weeks old were given an antibiotic cocktail of 0.1 mg/ml Rifampicin, 0.3 mg/ml Ampicillin and 2 mg/ml Streptomycin in their drinking water for 6 days to reduce endogenous gut bacteria. Subsequently, *E*. *coli* strains were provided in drinking water for 24 hours prepared from an over-day culture of OD_600nm_ 3 and/or anaerobic faecal culture grown to its maximum for 2 days, centrifuged at 4610 RCF for 5 minutes to collect bacteria and diluted 1:10 to obtain ~3 × 10^8^ bacteria/ml. The next day *P*. *aeruginosa* (strain PA14) was provided daily for 7 days in the drinking water as for *E*. *coli*. Then mice were injected intraperitoneally with 150 mg/kg of body weight with cyclophosphamide (CP) and 3 days later with another dose of 100 mg/kg as previously described^31^. Survival was observed twice a day until all mice die or for up to 1 week.

### Acid and sugar measurements

Lactic and acetic acid concentrations in culture supernatants and homogenized mouse faeces (produced via bead homogenization in water) were determined enzymatically using R-Biopharm kits No. 11112821035 and No. 10148261035 respectively, according to manufacturer’s instructions. Sugar concentrations in homogenized mouse faeces were determined using the Megazyme Sucrose/D-Fructose/D-Glucose Assay Kit (K-SUFRG) according to manufacturer’s instructions. Absorbance was measured using the NanoDrop 2000c Spectrophotometer.

### Pyocyanin measurement

Overnight PA14 cultures were diluted to OD_600nm_ 1, then 0.25 ml was used to inoculate 25 ml of LB. Cultures were grown at 37 °C, 200 rpm in 250 ml flasks. Supernatants were collected after centrifugation at 4800 RCF for 10 minutes. 4.5 ml of chloroform was added to 7.5 ml of supernatant and vortexed. Samples were then centrifuged at 4800 RCF for 10 minutes. 3 ml of the resulting blue layer at the bottom was transferred to a new tube. 1.5 ml of 0.2 M HCl was added to each tube and vortexed 2 times for 10 seconds. Samples were centrifuged for 3 minutes at 4800 RCF and 1 ml of the pink layer was transferred to cuvettes. Pyocyanin concentration (μg/ml) was calculated by multiplying the spectrophotometric measurements taken at OD_520nm_ by 17.072, then multiplying them again by 1.5 due to the chloroform dilution.

### Computational analysis

Pairwise comparisons of bacterial CFUs and other pairwise comparisons were evaluated using the two-sided Student’s t-test for samples of ≥10 and Mann–Whitney U-test or one-way analysis of variance (ANOVA) with post hoc Tukey’s multiple comparison test for samples <10. Survival curves of mice and flies were analysed with the Kaplan-Meier method and the log-rank test. All experiments were repeated at least twice with qualitatively similar results. Gene enrichment analysis was performed using the David’s functional annotation tool. Correlation coefficient (*R*) significance analyses of mouse faecal acid concentration vs. LT50 was done using Pearson correlation and an n = 6 (the average of six dietary conditions sampling 6 mice for each). The Acetic + Lactic acid Index for each of the 6 dietary conditions was computed by dividing each acid concentration of each dietary condition with the average concentration of that acid in all conditions and adding the normalized values of the two acids. For sucrose assimilation prediction we used BLASTN 2.8.1+ per Zhang *et al*. 2000^32^ and found (a) an *E*. *coli W* sucrose hydrolase (98% identity), (b) a sucrose permease (98% identity), (c) a sucrose-specific IIBC component (100% identity) and (d) a sucrose-6-phosphate hydrolase (100% identity) present in *E*. *coli* O127:H6 str. E2348/69 (taxid:574521), but not in the genomes of *E*. *coli* BW25113 (taxid:679895) and *E*. *coli* DH5[alpha] (taxid:668369).

## Results

### *Escherichia coli*-secreted factors antagonize *Pseudomonas aeruginosa* growth in the presence of sugars

**Figure 1:**
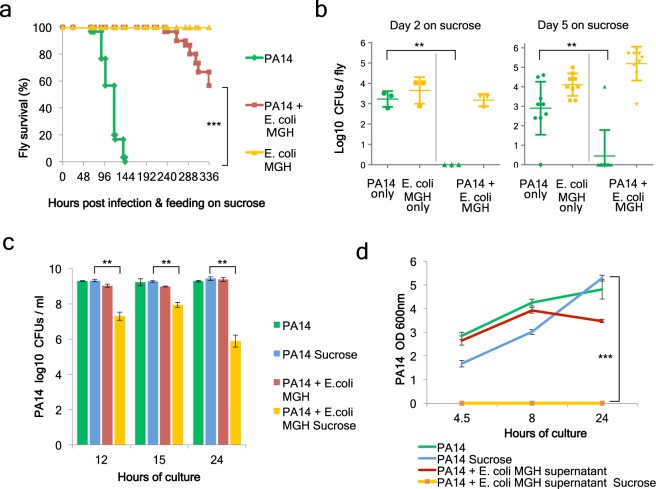
*E*. *coli* MGH inhibits *P*. *aeruginosa* growth and virulence in the *Drosophila* gut and in culture in the presence of sucrose. (**a**) Survival of *Drosophila melanogaster* Oregon R flies infected with PA14, *E*. *coli* strain MGH or co-infected with *E*. *coli* MGH and *P*. *aeruginosa* PA14 [n = 30]. (**b**) Colonization levels measured in colony forming units (CFUs) at Day 2 [n = 3] and Day 5 [n = 9] post-PA14-infection only, *E*. *coli* MGH only, and upon co-infection (triangles for PA14, inverted triangles for MGH). (**c**) CFUs of PA14 growth in the presence or absence of 4% sucrose and *E*. *coli* MGH in LB cultures [n = 3]. (**d**) Optical density measurements at 600 nm of PA14 growth in half fresh LB, half liquid supernatant of *E*. *coli* LB cultures + /− 4% sucrose [n = 9]. **p < 0.005,***p < 0.0005. Error bars represent standard deviation of the mean.

Screening for bacterial strains that may alleviate *P*. *aeruginosa* infection in *Drosophila*, we compared and combined the highly virulent *P*. *aeruginosa* strain, PA14, which kills all orally infected flies within 6 days^29^, with various *E*. *coli* strains (MGH, EPEC, BW25113 and DH5a), none of which was by itself significantly lethal to flies. The fly lethal time 50% (LT50%) extended beyond the 15 days for all *E*. *coli* strains, as exemplified with *E*. *coli* MGH and BW25113 shown in Fig. 1a and Suppl. Fig. 1a. Strikingly, *P*. *aeruginosa*-mediated fly lethality, fly colonization and *P*. *aeruginosa* growth in culture was dramatically inhibited by the human *E*. *coli* isolate MGH (Fig. 1a–d) and the laboratory *E*. *coli* strain BW25113 (Suppl. Fig. 1a–d) in the presence of 4% sucrose or 4% glucose, respectively. Noticeably, sucrose can be used by the *E*. *coli* strains MGH and EPEC to inhibit *P*. *aeruginosa* lethality and growth (fly LT50% > 10 days; Fig. 1a–d), because EPEC, for example, has 4 sucrose uptake and metabolism enzymes, namely, an *E*. *coli* W sucrose hydrolase, a sucrose permease, a sucrose-specific IIBC component and a sucrose-6-phosphate hydrolase. In contrast, the *E*. *coli* strains BW25113 and DH5a do not have these genes and were unable to utilize sucrose to inhibit *P*. *aeruginosa* in our experiments (fly LT50% < 7 days). As expected, when 4% glucose instead of sucrose was used in the infection mix, *E*. *coli* BW25113 gained the capacity to inhibit *P*. *aeruginosa* lethality, fly colonization and in culture growth (Suppl. Fig. 1a–d)^33^.

Of note, *E*. *coli* antagonizes *P*. *aeruginosa* not only in fly survival but also in inhibiting its colonization. We assessed the bacterial loads of each bacterial strain independently in the fly and upon co-infection. Colony forming unit (CFU) measurements in selective media revealed that at 2 days and 5 days after infection with either *P*. *aeruginosa* or *E*. *coli* MGH flies harboured roughly 10^3^ bacteria or more per fly (Fig. 1b). Upon co-infection with *E*. *coli* MGH, *P*. *aeruginosa* was almost eradicated, while *E*. *coli* MGH remained stable (Fig. 1b). Similarly, co-infection with the *E*. *coli* BW25113 strain and *P*. *aeruginosa* (PA14) resulted in lower PA14 CFUs and tentatively lower BW25113 CFUs at day 5 (Suppl. Fig. 1b), suggesting that mutual inhibition at the level of colonization is possible.

To assess if the inhibition between *P*. *aeruginosa* and *E*. *coli* is direct, we assessed bacterial growth in aerobic LB cultures. Interestingly, *E*. *coli* MGH did not inhibit *P*. *aeruginosa* growth in plain liquid LB (Fig. 1c). To assess if sucrose added in the fly infection media as a standard carbon source for the flies would make a difference in bacterial interactions in culture, we supplemented the LB media with 4% sucrose. Strikingly, in the presence of sucrose, *P*. *aeruginosa* CFUs were reduced by >1,000 fold when co-cultured with *E*. *coli* MGH, but no inhibition was noticed in the absence of sucrose (Fig. 1c). The monosaccharides glucose and fructose enable also the *E*. *coli* strain BW25113 to inhibit *P*. *aeruginosa* (Suppl. Fig. 1c). To assess whether secreted factors are responsible for *P*. *aeruginosa* growth inhibition we grew *P*. *aeruginosa* in a mix of 50% fresh LB and 50% filtered LB supernatant from an overnight *E*. *coli* culture that was supplemented or not with 4% sugar. The mix containing supernatant of *E*. *coli* MGH grown in sucrose and that of *E*. *coli* BW25113 grown in glucose was able to completely inhibit the growth of *P*. *aeruginosa* for at least 24 hours (Fig. 1d, Suppl. Fig. 1d).

### *E*. *coli* inhibits *P*. *aeruginosa* intestinal colonization and lethality during mouse gut-derived sepsis

To model the antibiotic-induced dysbiosis of mammals we used a mouse assay of intestinal infection. We administered a regime of three broad-spectrum antibiotics in mice and assessed their gut microbiota at the genus level through 16S sequencing analysis. In the absence of antibiotics, the microbiota consisted primarily of *Bacteroidetes*, *Firmicutes* and *Proteobacteria*, including *E*. *coli* (Suppl. Fig. 2a). Using colony PCR sequencing we verified the presence of an endogenous *E*. *coli* strain (naming the respective cultured strain CD1) and further identified 7 easy-to-culture and potentially beneficial strains belonging to the *Lactobaccillus*, *Bifidobacterium* and *Bacteroides* genera in the faeces of mice (Suppl. Fig. 2b). Antibiotic treatment induced dysbiosis, which is exemplified by the eradication of *E*. *coli*, the reduction of all the prevalent phyla below the detection level (Suppl. Fig. 2c), and the eradication of all 8 cultured bacterial strains except for *Bifidobacterium sp*.*2*, which was reduced from 8.4 log_10_ to 7.2 log_10_ CFUs per gram of mouse faeces (Suppl. Fig. 2d).

**Figure 2:**
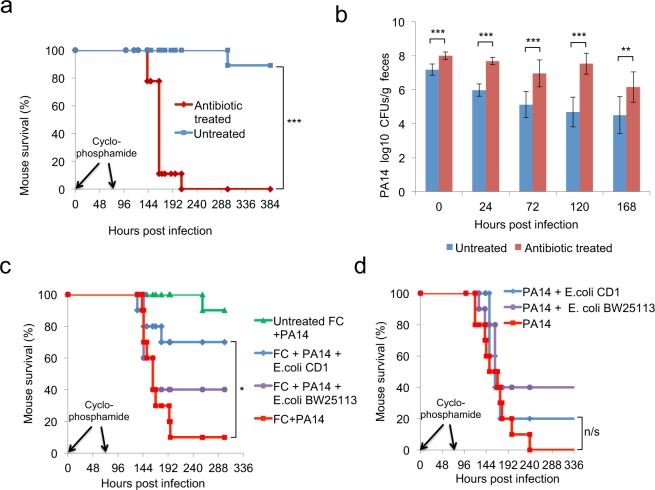
Commensal bacteria and *E*. *coli* protect antibiotic- and cyclophosphamide-treated mice from *P*. *aeruginosa*-induced lethality and colonization. (**a**) Survival to *P*. *aeruginosa* infection of immunocompromised mice pre-treated with antibiotics or untreated [n = 9]. (**b**) PA14 CFUs in faeces of immunocompromised mice pre-treated with antibiotics or untreated [n = 9]. (**c**) Survival to PA14 infection of immunocompromised mice feeding on a faecal culture (FC) fortified with *E*. *coli* BW25113 or mouse isolate *E*. *coli* CD1. Controls include antibiotic-treated and PA14-infected immunocompromised mice without *E*. *coli*, and mice without *E*. *coli* or antibiotic treatment [n = 10]. (**d**) Survival of antibiotic-treated PA14-infected immunocompromised mice feeding on the commensal mouse *E*. *coli* CD1 or the *E*. *coli* BW25113. Control immunocompromised mice were antibiotics-treated and infected with PA14, but no *E*. *coli* [n = 10]. n/s = p > 0.05, *p < 0.05, **p < 0.005, ***p < 0.0005. Error bars represent standard deviation of the mean.

Antibiotic-treated mice subjected to immunosuppression via cyclophosphamide injections and infected with *P*. *aeruginosa* exhibit systemic spread of bacteria (Suppl. Fig. 3a–c) and die from sepsis as previously described^31^. Notably, all immunosuppressed dysbiotic mice died within 9 days of oral infection with *P*. *aeruginosa*, while 90% of the *P*. *aeruginosa*-infected immunocompromised mice that are not treated with antibiotics survived (Fig. 2a). Accordingly, *P*. *aeruginosa* load in the stools of infected mice bearing the healthy microbiota were significantly less at all time points than in mice treated with antibiotics, suggesting that commensal microbes inhibit colonisation by *P*. *aeruginosa* (Fig. 2b). To partly re-establish the mouse microbiome, we administered a faecal culture supplement (FC) prepared from a pelleted anaerobic stool culture. FC contained the endogenous *Bacteroides*, *Bifidobacteria* and *Lactobacillus* species and to a lesser extent the endogenous *E*. *coli*. The addition of FC in the drinking water had little to no effect by itself in protecting mice against lethality. However, FC fortified with the endogenous *E*. *coli* strain (*E*. *coli* CD1) rescued 70% of mice (Fig. 2c). On the other hand, the *E*. *coli* CD1 in the absence of FC did not protect mice against *P*. *aeruginosa* infection (Fig. 2d), suggesting a synergism between the endogenous *E*. *coli* CD1 and other members of the microbiota as a result of adaptation or co-evolution. Unlike the *E*. *coli* CD1 strain, the laboratory *E*. *coli* strain BW25113 showed only a trend in improving mouse survival due to *P*. *aeruginosa* infection, and this effect was not modifiable by FC (Fig. 2c,d). Despite the marginal effect on survival, *E*. *coli* BW25113 can stably colonize the mouse gut (Suppl. Fig. 3d) and reduces the *P*. *aeruginosa* burden significantly in the mouse gut within a week post-infection (Suppl. Fig. 3e).

### Aerobic or anaerobic fermentation of glucose to lactic and acetic acid by *E*. *coli* is necessary for inhibiting *P*. *aeruginosa* growth

**Figure 3:**
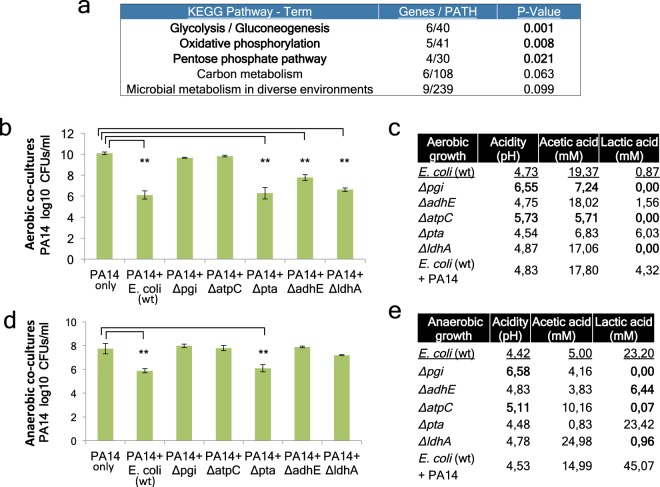
*E*. *coli* mutants deficient in inhibiting *P*. *aeruginosa* and their acetic and lactic acid production profiles. (**a**) Enrichment analysis of *E*. *coli* genes identified as necessary for PA14 inhibition using EASE Score, a modified Fisher Exact P-Value. (**b**,**d**) PA14 CFUs in co-cultures with *E*. *coli* BW25113 (WT) and isogenic gene mutants at 5 hours of aerobic growth (**b**) or 24 hours of anaerobic growth (**d**) [n = 6]. (**c**,**e**) Liquid culture media pH and acid concentration (mM) at 5 hours of aerobic growth (c) or 24 hours of anaerobic growth (**e**) [n = 6]. Bold values indicate deviation from the wild type of mutant *E*. *coli* strains. **p < 0.005. Error bars represent standard deviation of the mean.

*E*. *coli* QS signalling and the production of the metabolite indole have been reported to inhibit *P*. *aeruginosa* growth^27,34^. To reveal *E*. *coli* factors that inhibit *P*. *aeruginosa* in our glucose-supplemented media, we assessed *E*. *coli* QS mutants and indole production genes previously implicated in bacterial competition^27^. We found that the *E*. *coli* QS genes *luxS* and *sdiA* are not necessary for *P*. *aeruginosa* inhibition in an LB culture supplemented with 4% glucose (Suppl. Fig. 4a). In addition, a deletion of the indole production enzyme tryptophanase (*tna*) essentially eliminated indole production (Suppl. Fig. 4b), but not the ability of *E*. *coli* BW25113 to inhibit *P*. *aeruginosa* (Suppl. Fig. 4c). Therefore we performed an unbiased screen of the KEIO collection of 3985 isogenic K-12 BW25113 gene mutants, identifying 45 genes that are necessary for the inhibition of *P*. *aeruginosa* in LB broth supplemented with 4% glucose. Gene enrichment analysis pinpointed glycolysis and the downstream pathways of oxidative phosphorylation and pentose phosphate as strongly enriched (Fig. 3a).

To assess the impact of *E*. *coli* glycolysis and oxidative phosphorylation on *P*. *aeruginosa* growth, we co-cultured *P*. *aeruginosa* with the core glycolysis and oxidative phosphorylation pathway mutants of *E*. *coli*, *Δpgi* and *ΔatpC*, respectively. In aerobic cultures using LB plus 4% glucose, wild-type *E*. *coli* BW25113 reduced *P*. *aeruginosa* CFUs by >1,000 fold, while *Δpgi* and *ΔatpC* mutants were unable to inhibit *P*. *aeruginosa* growth significantly (Fig. 3b). This is in line with the fermentation efficiency of the *Δpgi* and *ΔatpC* strains, which was severely compromised with no lactic acid and reduced acetic acid (>2 fold decrease) production and deficient acidification (pH > 5.5) of the liquid bacterial culture (Fig. 3c). In aerobic conditions lactic acid production is very low compared to acetic acid production, but none of the mixed acid fermentation mutants, *Δpta*, *ΔadhE* or *ΔldhA*, could abolish production of lactic acid and reduce acetic acid production at the same time (Fig. 3c). Accordingly, these mutants retained their ability to inhibit *P*. *aeruginosa* aerobically (Fig. 3b). On the other hand, the *Δpgi* and *ΔatpC* strains abolish lactic acid and reduce acetic acid production, and these mutants are the only ones unable to inhibit *P*. *aeruginosa* (Fig. 3b,c).

Because the environment in the mammalian gut is anaerobic and the fermentation process towards lactic acid production is much more efficient, we further tested this pathway anaerobically. As under aerobic conditions, the core metabolism *E*. *coli* mutants *Δpgi* and *ΔatpC* were unable to inhibit *P*. *aeruginosa* growth, acidify culture media and produce lactic acid in anaerobic cultures (Fig. 3d,e). Also *E*. *coli ΔldhA* and *ΔadhE* mutants exhibited significantly reduced lactic acid production [P < 0.001] (Fig. 3e) and an impaired ability to inhibit *P*. *aeruginosa* in an anaerobic culture (Fig. 3d). Thus, lactic acid production is crucial, while acetic acid production is helpful, in inhibiting *P*. *aeruginosa* growth either aerobically or anaerobically.

### Lactic acid and acetic acid can inhibit *P*. *aeruginosa* growth and virulence

**Figure 4:**
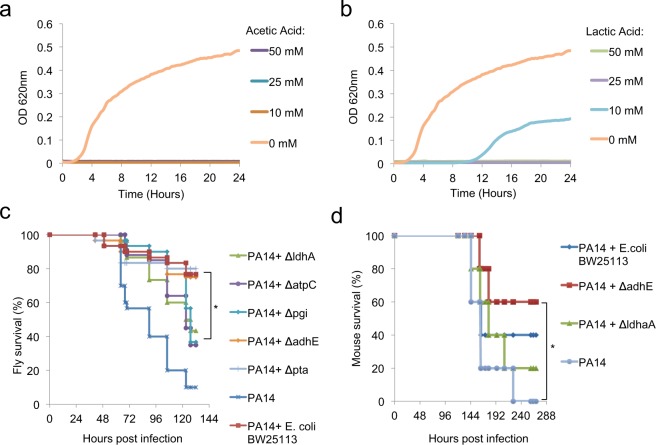
Lactic and acetic acid capacity to inhibit *P*. *aeruginosa* growth in culture media and virulence in flies and mice. (**a**,**b**) PA14 growth in LB broth supplemented with 0, 10, 25 and 50 mM of acetic acid (**a**) or lactic acid (**b**) at pH 5 [n = 4]. (**c**) *Drosophila* survival curves upon co-infection with PA14 and wild-type (BW25113) or isogenic mutant *E*. *coli* strains [n = 30]. (**d**) Survival of PA14-infected immunocompromised mice complemented with a faecal culture (FC) and wild-type *E*. *coli* BW25113 or *E*. *coli* mutants, *ΔldhA* or *ΔadhE*, or no *E*. *coli* [n = 10]. *p < 0.05.

Supplementation of the *E*. *coli* mixed-acid fermentation products acetic acid and lactic acid have been reported to act as antimicrobials against *P*. *aeruginosa*^35–37^. We validated the role of these two metabolites in inhibiting *P*. *aeruginosa* growth at pH 5. Acidic pH of <5 is observed in an *E*. *coli* culture in the presence of sugars in either aerobic or anaerobic conditions (Fig. 3c,e). A concentration of 10 mM or more of acetic acid, which can be produced by *E*. *coli* in an aerobic liquid culture (Fig. 3c), abolished *P*. *aeruginosa* growth (Fig. 4a). Similarly, 10 mM or more of lactic acid, which can be produced by *E*. *coli* in an anaerobic culture (Fig. 3e), inhibited *P*. *aeruginosa* growth (Fig. 4b).

The inhibitory effect of *E*. *coli* lactic and acetic acid genes was further tested in animal models. In fly infection experiments, the *ΔldhA E*. *coli* mutant, specifically deficient in lactic acid production, and the core metabolism mutants *Δpgi* and Δ*atpC*, which are also unable to produce lactic acid aerobically or anaerobically, exhibited diminished ability to rescue flies infected with *P*. *aeruginosa* (Fig. 4c). In contrast, the Δ*pta* and Δa*dhE* mutants, which cannot abolish lactic acid production in culture, rescued flies to the levels of the wild-type isogenic *E*. *coli* strain ΒW25113 (Fig. 4c). The same pattern was observed during co-infections in mice. We noticed that the *E*. *coli* mutant *ΔadhE* significantly rescued 60% of mice from lethality upon oral *Pseudomonas* infection in mice, comparable to the wild-type isogenic *E*. *coli* strain ΒW25113 that rescued 40% of mice, unlike the lactic-acid-defective strain Δ*ldhA* that did not provide any significant rescue (Fig. 4d), suggesting that lactic acid production is the key for *E*. *coli* to inhibit *P*. *aeruginosa* in the host.

### *P*. *aeruginosa* antagonizes *E*. *coli* strains unable to ferment sugars to lactic acid

**Figure 5:**
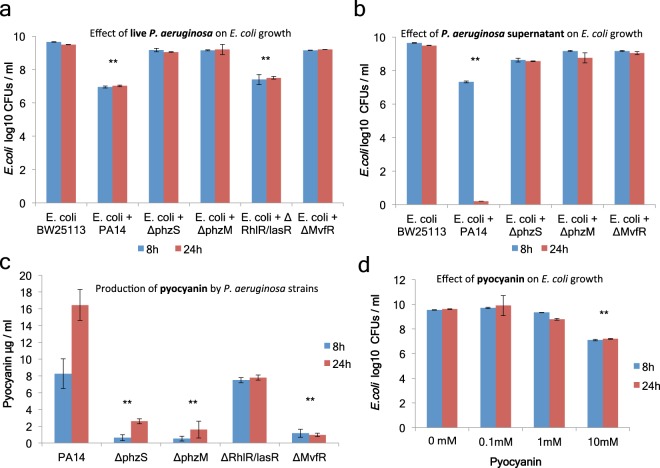
*P*. *aeruginosa* toxin pyocyanin inhibits *E*. *coli* growth in culture. (**a**) CFUs of *E*. *coli* BW25113 in co-culture with wild-type PA14 vs. isogenic QS mutants at 8 and 24 hours [n = 6]. (**b**) CFUs of *E*. *coli* BW25113 in 1:1 LB to LB supernatant of PA14 vs. isogenic QS mutants at 8 and 24 hours [n = 6]. (**c**) Pyocyanin concentration in LB cultures of wild-type PA14 vs. isogenic QS mutants at 8 and 24 hours [n = 6]. (**d**) CFUs of BW25113 at 8 and 24 hours in LB with supplemented pure pyocyanin [n = 6]. **p < 0.005. Error bars represent standard deviation of the mean.

In the absence of added sugars in the culture media, *P*. *aeruginosa* inhibited *E*. *coli* growth rather than being inhibited by it (Fig. 5a,b). Screening for *P*. *aeruginosa* mutants implicated in this process, we identified the phenazine system and its known regulators (*phzS*, *phzM* and *mvfR*) as necessary for *E*. *coli* growth inhibition by >100 fold in culture (Fig. 5a,b). Pyocyanin, a redox-active secondary metabolite and a potent antibacterial, is produced and secreted by *P*. *aeruginosa* under the strict control of these quorum-sensing regulators. On the other hand the lasR/rhlR QS system regulators cannot fully control the expression of pyocyanin (Fig. 5c), and thus their mutation does not abolish the ability of *P*. *aeruginosa* to inhibit *E*. *coli* (Fig. 5a). Supplementation of 10 mM of pure pyocyanin was sufficient to inhibit *E*. *coli* in LB cultures to the same extent as in co-cultures with *P*. *aeruginosa* (Fig. 5a,d).

**Figure 6:**
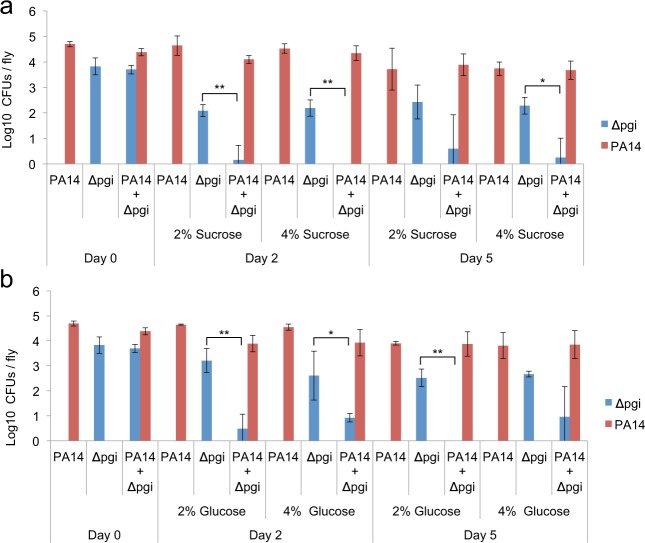
*P*. *aeruginosa* inhibits fermentation mutant *E*. *coli* in the *Drosophila* gut, despite dietary sucrose or glucose. (**a**,**b**) *Drosophila* intestine CFUs at 0, 2 and 5 days of flies orally infected with the *E*. *coli* BW25113 *Δpgi* core glycolysis mutant and/or PA14 [n = 6]. 2% or 4% sucrose (**a**) or glucose (**b**) was added in the infection medium and the fly food as a standard nutrient for the flies. *p < 0.05, **p < 0.005. Error bars represent standard deviation of the mean.

Moreover, the *E*. *coli* mutants, *Δpgi* and *Δldha*, which are deficient in glycolysis and lactic acid production respectively, were unable to inhibit *P*. *aeruginosa* growth in culture with and without supplementation of 4% glucose or sucrose (Suppl. Fig. 5a,b). Accordingly, we assessed the ability of *P*. *aeruginosa* to inhibit colonization by the *E*. *coli* mutants *Δpgi* and *Δldha*. Flies were inoculated with one or both species together and offered 2% or 4% glucose or sucrose as a necessary fly food nutrient. Co-inoculation of *Drosophila* with the *E*. *coli* glycolysis mutant *Δpgi* and *P*. *aeruginosa* showed reduced *E*. *coli* CFUs on day 2 and 5 compared to single inoculation with *E*. *coli Δpgi* (Fig. 6a,b). Moreover, flies mono-inoculated with *P*. *aeruginosa* or the *E*. *coli* lactic acid mutant *Δldha* could be colonized with an average of 4.1 (±0.5 SD) and 3.5 (±0.2 SD) log_10_ CFUs, respectively. Immediately after co-inoculation though *E*. *coli Δldha* CFUs were low, exhibiting an average of 1.9 (±0.2 SD) log_10_, while *P*. *aeruginosa* remained high at 3.8 (±1 SD) log_10_ CFUs. Thus with or without dietary sugars *P*. *aeruginosa* may inhibit gut colonization by *E*. *coli* mutants unable to ferment sugars into lactic acid.

### Lactic and acetic acid production rather than nutritional input defines the interaction between *E*. *coli* and *P*. *aeruginosa* in the mouse gut

**Figure 7:**
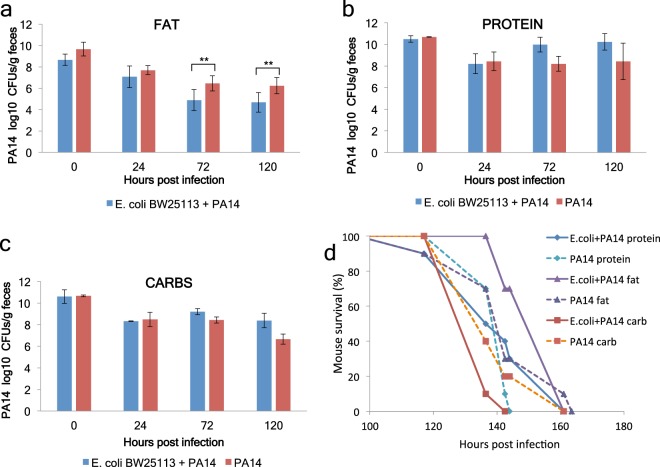
A fat-based diet, but not a carb- or protein-based diet enables *E*. *coli* to inhibit *P*. *aeruginosa* colonization and lethality in mice. (**a**) PA14 CFUs in faeces of immunocompromised mice fed on a fat- (**a**), protein- (**b**) or carbohydrate-based diet following inoculation with PA14 or co-inoculation with PA14 and *E*. *coli* BW25113 [n = 10]. **p < 0.005. Error bars represent standard deviation of the mean. (**d**) Survival of immunocompromised mice kept on a fat-, protein-, or carb-based diet and infected with PA14 or PA14 plus *E*. *coli* BW25113 [n = 10]. p = 0.04 for Fat PA14 + *E*. *coli* vs. Protein PA14 + *E*. *coli* and p = 0.0001 for Fat PA14 + *E*. *coli* vs. Carbs PA14 + *E*. *coli*.

**Figure 8:**
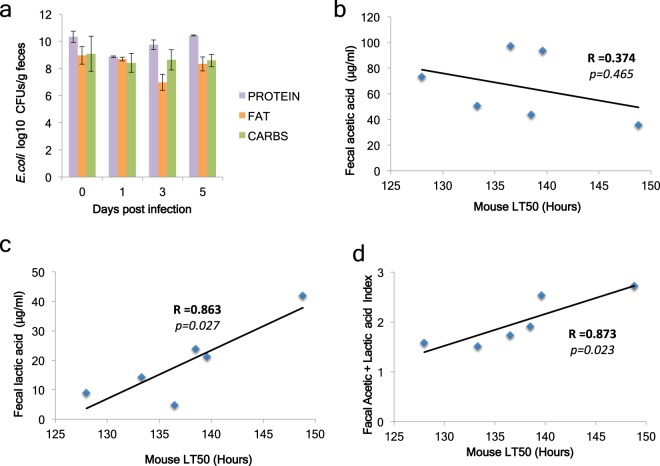
Faecal concentration of lactic and acetic acid correlates with mouse survival upon *P*. *aeruginosa* regardless of faecal *E*. *coli* levels. (**a**) *E*. *coli* CFUs in faeces of immunocompromised mice infected with PA14 and *E*. *coli* BW25113 and kept on a protein-, fat- or carbohydrate-based diet [n = 10]. (**b**,**c**) Correlation plots and Pearson correlation coefficient (R) of faecal acetic acid (**b**) and lactic acid (**c**) concentration against the lethal time 50% (LT50) of the corresponding mice for each of the 6 conditions (3 mouse diets × 2 types of infection). (**d**) A combinatorial index of normalized mouse faecal acetic and lactic acid concentration correlated with LT50.

Diet is very important for the maintenance of a healthy microbiome and in shaping the intestinal immune response^38–40^. Our study shows that the interaction between *E*. *coli* and *P*. *aeruginosa* is shaped by the fermentation of sugars. Therefore we sought to investigate in mice the contribution of three nutritionally extreme diets: a protein-based, a vegetable fat-based and a carbohydrate-based diet. In mice on the carbohydrate-based diet the total sugar concentration (sucrose, glucose and fructose) in the faeces was 67.4 μg/ml, which was higher than any of the other diets, while it was only 18.7 μg/ml in the faeces of mice given *E*. *coli* orally (Suppl. Fig. 6a). This means that *E*. *coli* is consuming sugars in the mouse gut. Yet faecal lactic acid concentration was the highest in the fat-based diet group in the presence of *E*. *coli* (Suppl. Fig. 6b). Accordingly, *P*. *aeruginosa* CFUs were reduced by *E*. *coli* in mice fed with the vegetable fat-based diet (Fig. 7a), but not with the protein- or the carbohydrate-based diets (Fig. 7b,c). Similarly, mouse survival upon *P*. *aeruginosa* infection in immunosuppressed mice was the highest in mice fed the vegetable fat-based diet and co-inoculated with *E*. *coli*, as opposed to mice fed with the carbohydrate-based diet and co-inoculated with *E*. *coli* (Fig. 7d). However, the fat-based diet does not favour *E*. *coli* gut colonization, as the *E*. *coli* CFUs in the faeces are comparable between the carbohydrate- and the fat-based diets and lower than those of the protein-based diet (Fig. 8a).

To assess if faecal acetic and lactic acid production may indicate protection against *P*. *aeruginosa*, we correlated acid concentration in the faeces with the lethal time 50% (LT50) of the corresponding sets of mice. We found that lactic but not acetic acid levels alone correlate significantly and positively with survival to infection (Fig. 8b,c), while an index of normalized concentration values for acetic and lactic acid combined gave also a clear correlation with survival (Fig. 8d). We conclude that the standard balanced diet (Fig. 4d; Suppl. Fig. 3e) and the vegetable fat-based diet (Fig. 7a), rather than the carbohydrate-based diet (Fig. 7c) facilitate the inhibitory effect of *E*. *coli* on *P*. *aeruginosa*; and we suggest that, given the complexity of the mammalian intestinal environment, the metabolic output in acetic and lactic acid production rather than the dietary input is indicative of susceptibility to intestinal *P*. *aeruginosa* infection.

## Discussion

Despite primarily observations on bacterial antagonism dating more than 100 years ago^41^, there are not many cases supporting the model of one-pathogen-one-colonization-resistor, according to which specific bacterial strains protect the host against infection^42^. Known cases include the inhibitory effect of *Clostridium scindens* in resistance against *C*. *difficile* infection^43^, the non-toxigenic *Bacteroides fragilis* resistance against the enterotoxigenic *Bacteroides fragilis*^44^, the effect of *E*. *coli* O21:H+ against muscle atrophy due to infection, and the effect of *E*. *coli* EcN against intestinal pathogens^42^. Antagonistic interactions between a pathogenic and a non-pathogenic bacterial strain may include: (a) the direct inhibition of pathogen’s growth, colonization or virulence by the non-pathogenic strain, or (b) the indirect effect of the non-pathogenic strain in inducing or supporting the host defence to infection. To establish the mode of interaction between *E*. *coli* and *P*. *aeruginosa* we first examined whether *Drosophila* can be efficiently colonized with these species. Sugar-based diets allow stable bacterial colonization with either *P*. *aeruginosa* or *E*. *coli* strains. When flies are infected with both species, *P*. *aeruginosa* colonization and mortality is significantly reduced or eliminated. Antagonism between *P*. *aeruginosa* and other species is nevertheless specific. Human oropharyngeal bacteria are predominantly gram-positive, such as the *Neisseria*, *Streptococcus*, *Staphylococcus* and *Actinomyces* species, differ from those of the human intestine, and tend to induce rather than antagonize *P*. *aeruginosa* virulence^45^. Moreover, peptidoglycan, which is abundant in gram-positive bacteria, can directly induce the virulence of *P*. *aeruginosa*^46^. Thus *E*. *coli*, as opposed to many gram-positive bacteria, might serve as a safer inhibitor of *P*. *aeruginosa* by inhibiting its growth without inducing its virulence.

*P*. *aeruginosa* usually affects hospitalized and immunocompromised individuals. It causes life-threatening burn wound and lung infections, but humans often carry *P*. *aeruginosa* asymptomatically in their intestines^47^. During *P*. *aeruginosa* intestinal colonization, a healthy host primarily deploys innate immune responses recruiting macrophages and monocytes in the gut and then adaptive immune cells, such as B lymphocytes, through the induction of pro-inflammatory cytokines to control infection^48^. In immunocompromised patients, however, *P*. *aeruginosa* may disrupt the intestinal epithelial barrier and translocate extraluminally, leading to sepsis and death^49^. Moreover, virulent *P*. *aeruginosa* may facilitate this process by subverting the innate immune responses upon infection^50^. Another reason for the benignity of *P*. *aeruginosa* in the healthy human gut may be the action of intestinal microbiota, which are part of the host defence to intestinal infection^51–53^. Previous studies describe the use of antibiotic cocktails that favour *P*. *aeruginosa* intestinal colonization by compromising resistance by the intestinal microbiota^54^. Accordingly, we show that antibiotic use in mice diminishes all the prevalent phyla, eradicates *E*. *coli*, and induces dysbiosis. Using a *Pseudomonas*-induced gut-derived sepsis model to investigate infection in mice that exhibit neutropenia, lymphopenia, as well as mucosal damage^55,56^, we found that mice not given antibiotics mostly survived and were less colonized with *P*. *aeruginosa*, contrary to antibiotic-treated mice. In addition, reintroduction of the commensal microbes through a faecal culture of endogenous and potentially beneficial bacterial species was inefficient in improving mouse protection from lethality. Nevertheless, a high dose of the endogenous *E*. *coli* CD1 isolate in combination with faecal bacteria exhibited significant protection against *P*. *aeruginosa*. We postulate a symbiotic adaptation of the mouse-isolated *E*. *coli* strain with the mouse gut environment and its microbiota in protecting the host.

Contrary to a previous study^27^, we found that *E*. *coli* indole production had no effect on inhibiting the growth of *P*. *aeruginosa* in our experiments. This might be due to the inhibition of *E*. *coli* indole production by sugars added in our media or indole degradation via a higher induction of QS in the *P*. *aeruginosa* strain PA14^27,57^. Accordingly, we screened in an unbiased way and pinpointed *E*. *coli* glucose metabolism and fermentation mutants deficient in lactic and acetic acid production responsible for inhibiting *P*. *aeruginosa*. In antibiotic-treated mice, a similar trend was observed whereby the lactate-dehydrogenase-deficient *E*. *coli* mutant was unable to protect mice from *P*. *aeruginosa* infection and mortality. The anti-infective properties of lactic and acetic acid may be attributed to lowering the pH, but also to the permeabilization of the outer membrane of gram-negative bacteria^37^. On the other hand, *P*. *aeruginosa* produces many virulence factors regulated by QS, such as pyocyanin, which has bactericidal properties^58^. Accordingly, we notice that only strains of *P*. *aeruginosa* able to produce pyocyanin can inhibit *E*. *coli* unable to produce lactic or acetic acid due to the lack of sugars in the media. Interestingly, despite the fact that high sugar concentrations may inhibit *P*. *aeruginosa* QS^59^, *P*. *aeruginosa* grown in sugar-supplemented media can still inhibit *E*. *coli* strains with mutated fermentation pathway genes. Thus depending on the concentration of *E*. *coli*’s acetic and lactic acid or *P*. *aeruginosa’*s pyocyanin, the antagonistic growth may be shifted towards one or the other species.

The role of diet has been extensively studied in response to gut microbiota and host physiology^60^. Hence we explored three different diets, based either on carbohydrates (corn starch and sucrose), fat (vegetable shortening) or protein (casein). Mice feeding on these diets exhibited complex features: First, the carbohydrate-based diet did not improve the ability of *E*. *coli* to inhibit *P*. *aeruginosa* colonization and concomitant mortality. This might be because this carbohydrate-based diet does not deliver a significant amount of free sugars in the mouse colon. While sugars are higher in the faeces of mice fed with a carbohydrate-based diet, they may be too low to have the anticipated impact on *E*. *coli*. Second, the protein-based diet sustains more *E*. *coli* than the other diets, yet this didn’t translate into better inhibiting capacity against *P*. *aeruginosa*. This might be because casein inhibits or lacks the ability to fuel fermentation into lactic acid. Third, the vegetable fat-based diet, while not ideal for *E*. *coli* growth compared to the other diets, allows *E*. *coli* to produce more lactic acid that can inhibit *P*. *aeruginosa* growth. This is in line with evidence showing that unsaturated fat may benefit lactic acid bacteria in mice^61^.

Lactic acid in the mouse faeces is much lower than the lowest inhibitory concentration tested in culture. Nevertheless, *E*. *coli* mutants defective in lactic acid production are also defective in inhibiting *P*. *aeruginosa* in the fly and mouse gut. The ability of any chemical to inhibit bacterial growth depends on the environment and thus additional factors (e.g. additional antimicrobials or lactic acid metabolism products) in the fly and mouse gut may boost the ability of lactic acid to inhibit *P*. *aeruginosa*. In addition, high sugar levels may be difficult to achieve by a high carbohydrate diet because sugar is readily absorbed in the small intestine, and *E*. *coli* and other commensals may use dietary fat more efficiently towards lactic acid production. Thus the metabolic output in the colon rather than the dietary input might better dictate the balance between and among bacterial species. Metabolic output is nevertheless a result of diet, microbiota composition and the host physiology acting in concert. Accordingly, faecal metabolomics might prove very helpful in predicting the outcome of bacterial interactions in the human colon and the risk for an infection.

## Supplementary information

Suppl. Figures 1–6

## Data Availability

All data will be available upon publication.
