# Supplementary material for: Metabolic output defines Escherichia coli as a health-promoting microbe against intestinal Pseudomonas aeruginosa
Source: Sci Rep. 2019 Oct 8;9:14463. doi: 10.1038/s41598-019-51058-3 (PMC6783455; doi:10.1038/s41598-019-51058-3)
Supplement: Supplementary file 1 — Suppl. Figures 1–6 [file 41598_2019_51058_MOESM1_ESM.pdf]

Supplementary Information
Supplementary Figures for manuscript titled:

Metabolic output defines *Escherichia coli* as a health-promoting microbe against intestinal *Pseudomonas aeruginosa*

by

Theodoulakis Christofi[1], Stavria Panayidou[1], Irini Dieronitou[1], Christina Michael[1] & Yiorgos Apidianakis[1]*

1Department of Biological Sciences, University of Cyprus, Nicosia, Cyprus

*Corresponding author, email: apidiana@ucy.ac.cy

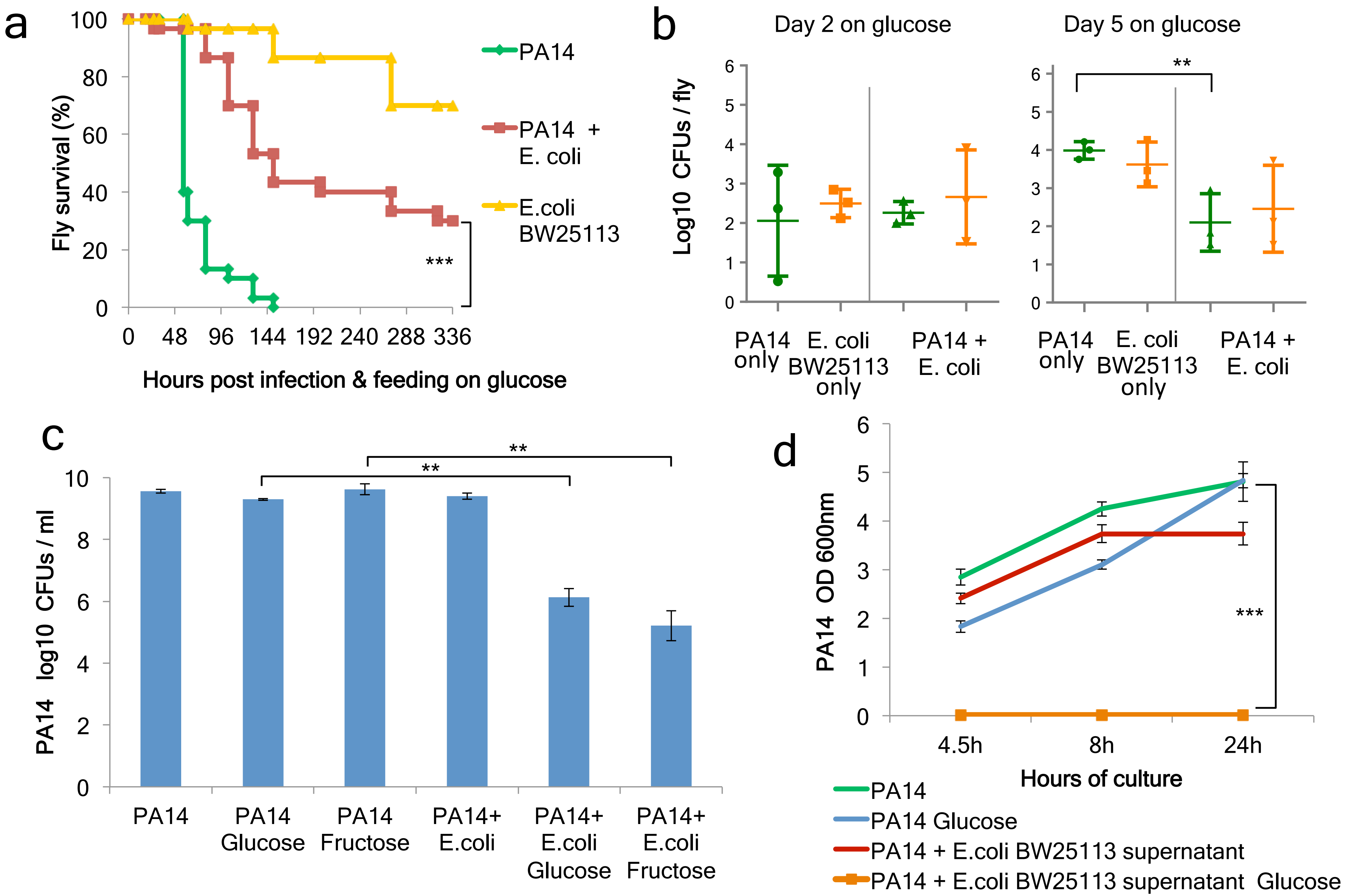

Suppl. Figure 1: *E. coli* BW25113 inhibits *P. aeruginosa* growth and virulence in the *Drosophila* gut and in culture in the presence of glucose. (a): Survival of *Drosophila* infected with *P. aeruginosa* strain PA14, *E. coli* strain BW25113, and upon co-infection (triangles for PA14, inverted triangles for BW25113) [n=30]. (b): CFUs at days 2 and 5 post infection with PA14 only, *E. coli* BW25113 only, or in mono or co-infected flies [n=3]. (c): CFUs of PA14 growth in the presence or absence of 4% glucose or 4% fructose and *E. coli* BW25113 in LB cultures [n=3]. (d): Optical density measurements at 600nm of PA14 growth in half fresh LB, half liquid supernatant of *E. coli* LB cultures +/- 4% glucose [n=9]. Error bars represent standard deviation of the mean.

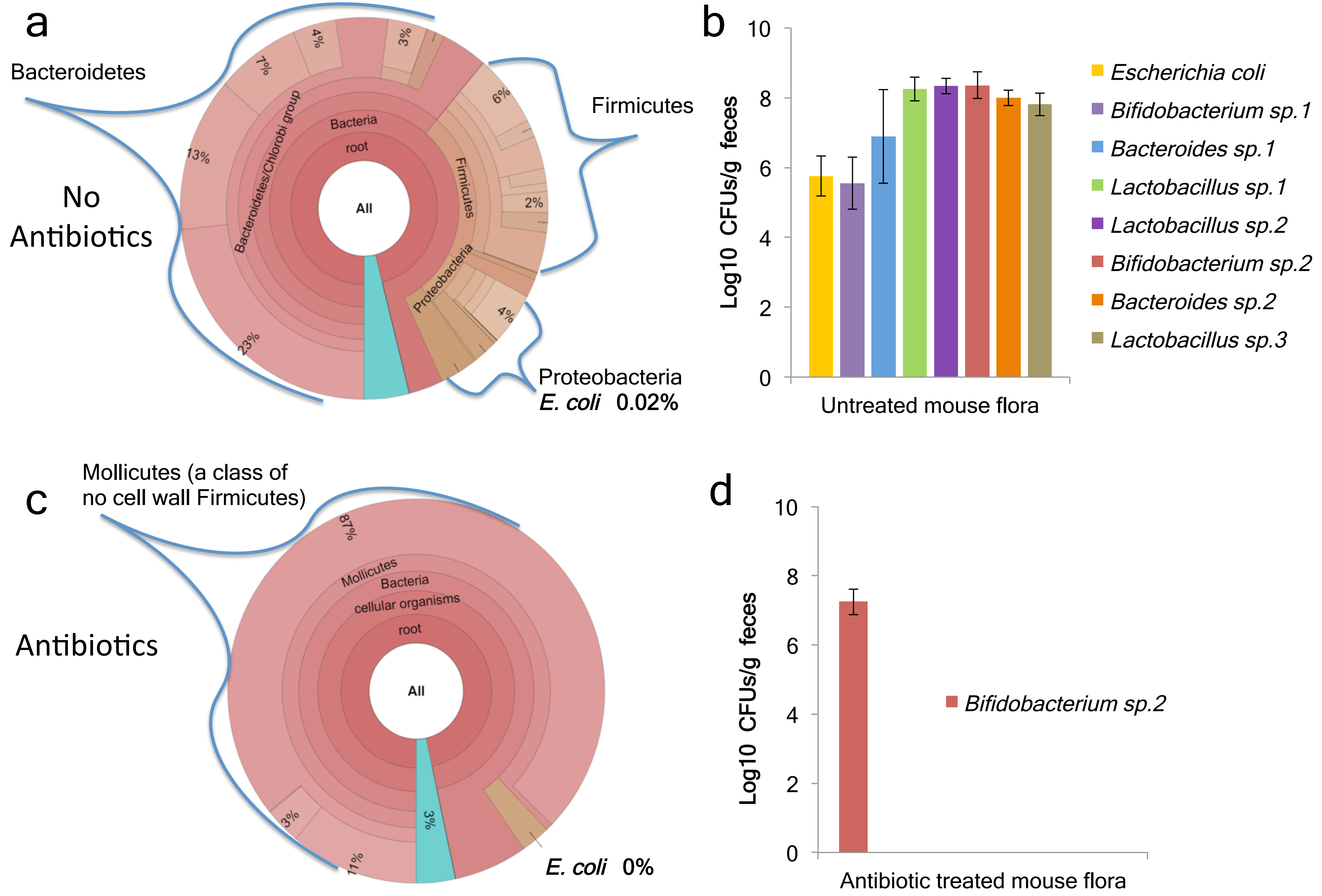

Suppl. Figure 2: Antibiotics eradicate potentially beneficial bacteria in the mouse feces. (a,b): 16S Metagenomics analysis of mouse feces before (a) and one week after (b) antibiotic treatment at the bacterial phylum level and the *E. coli* species level. (b,c): CFUs of cultured potentially beneficial bacteria in the feces of untreated mice (b) or mice treated with antibiotics (c) [n=7-9].

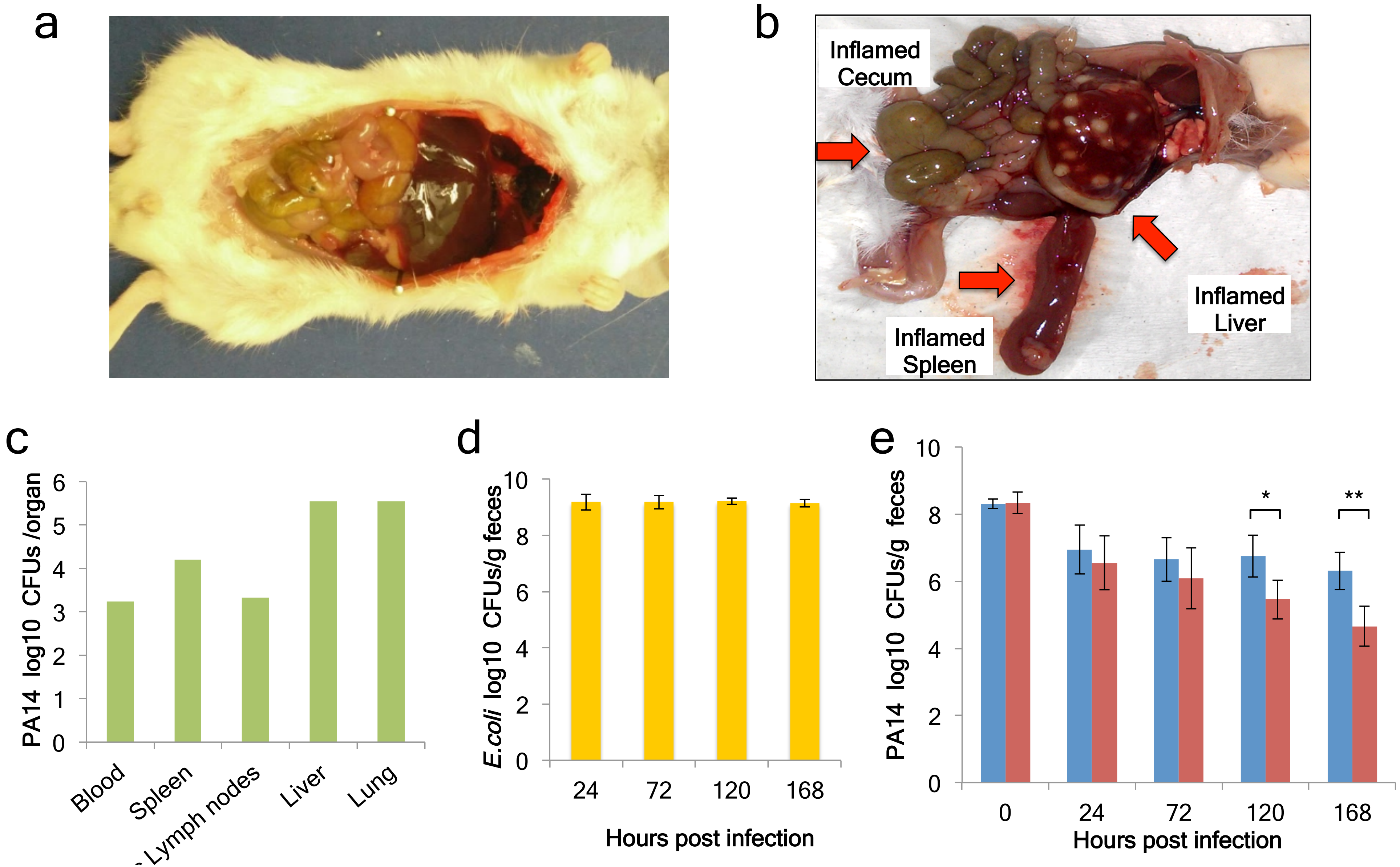

Suppl. Figure 3: *E. coli* colonisation and *P. aeruginosa* infection in mice immunocompromized through cyclophosphamide. (a): Internal organs of an untreated mouse. (b): Internal organs of an immunocompromized and antibiotic-treated and infected mouse. (c): PA14 log10 CFUs per ml of blood or whole organ counts in spleen, liver, lung and mesenteric lymph nodes. (d): *E. coli* BW25113 colonisation levels in mice infected for 7 Days with PA14 followed by 1 Day with *E. coli* at a concentration of 3x10^8 bacteria in the drinking water. (e): PA14 CFUs in feces of mice after antibiotic treatment and further supplementation with *E. coli* for 1 day. (d,e) Time points for sample measurements start after 7 days of PA14 infection in the drinking water [n=6]. *=p<0.05, **=p<0.005. Error bars represent standard deviation of the mean.

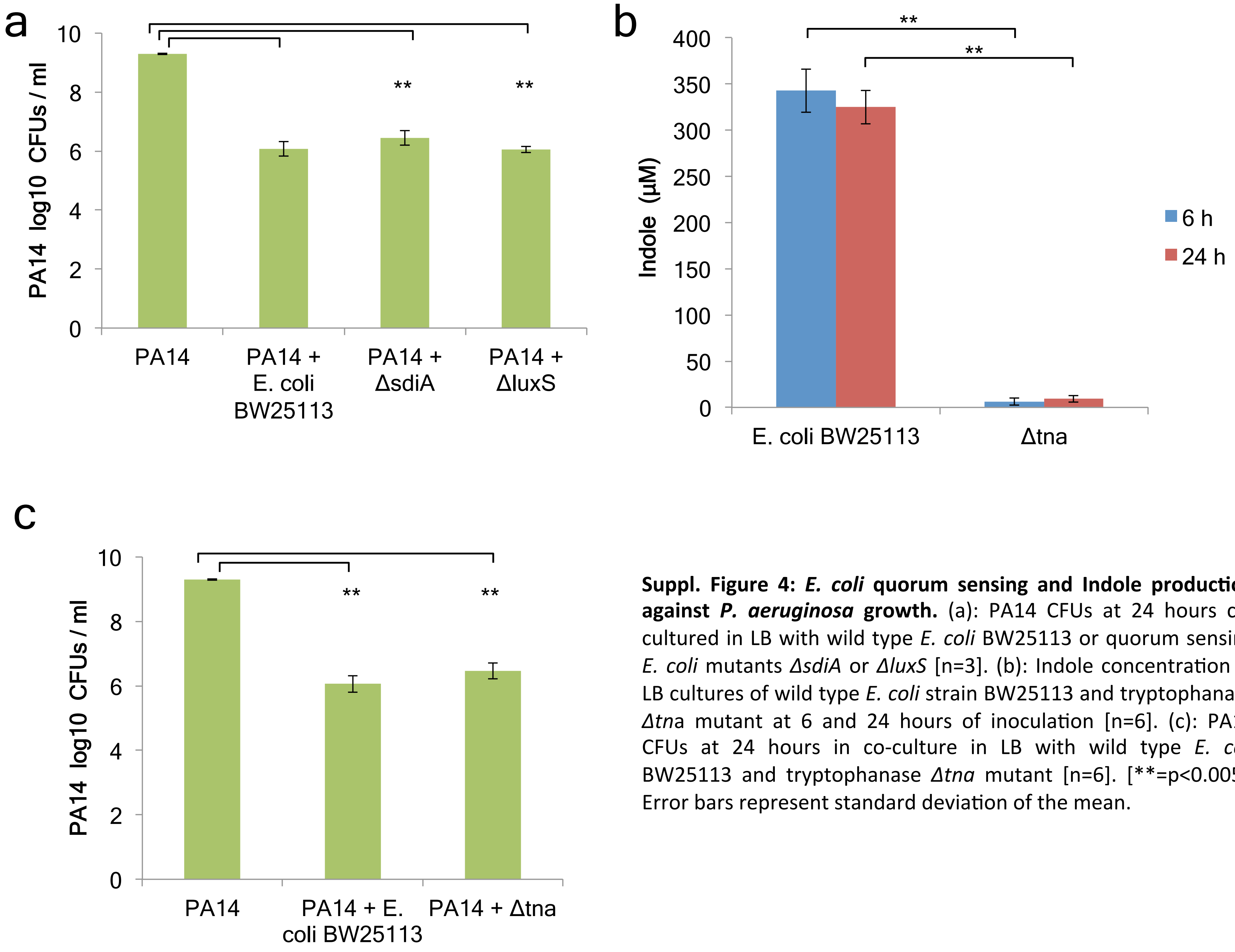

Suppl. Figure 4: *E. coli* quorum sensing and Indole production against *P. aeruginosa* growth. (a): PA14 CFUs at 24 hours co-cultured in LB with wild type *E. coli* BW25113 or quorum sensing *E. coli* mutants *ΔsdiA* or *ΔluxS* [n=3]. (b): Indole concentration in LB cultures of wild type *E. coli* strain BW25113 and tryptophanase *Δtn*a mutant at 6 and 24 hours of inoculation [n=6]. (c): PA14 CFUs at 24 hours in co-culture in LB with wild type *E. coli* BW25113 and tryptophanase *Δtna* mutant [n=6]. [**=p<0.005]. Error bars represent standard deviation of the mean.

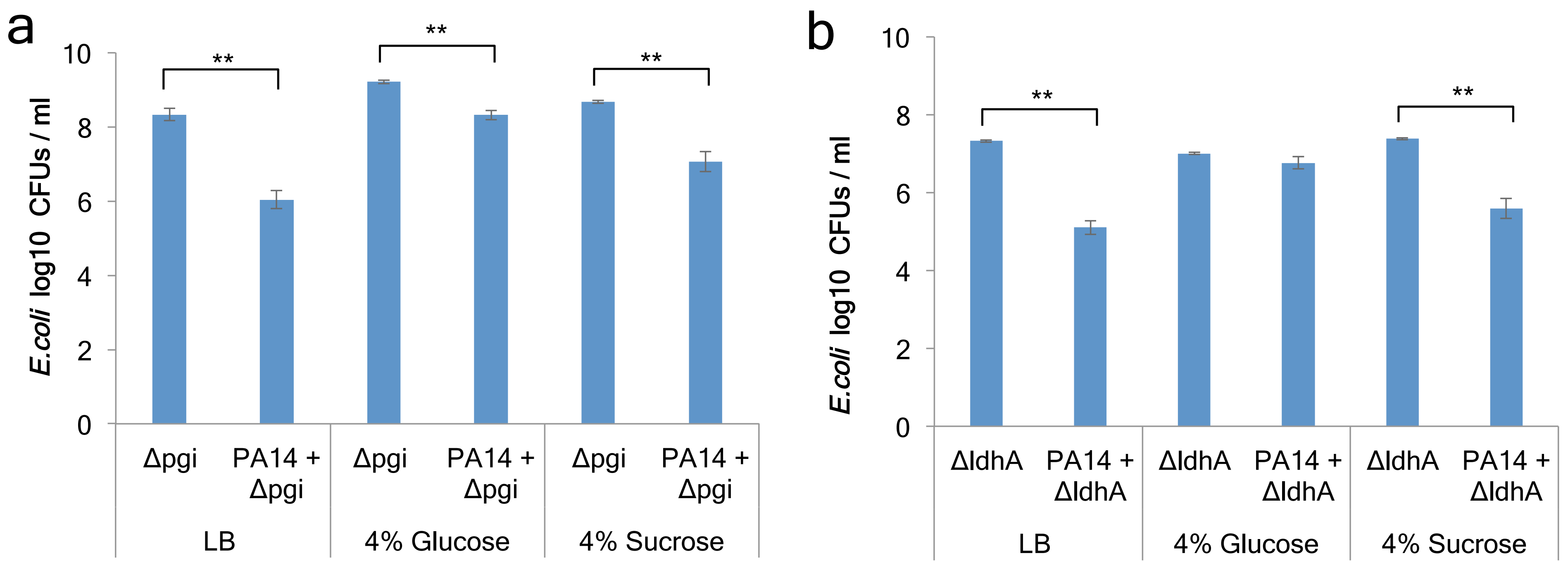

Suppl. Figure 5: Growth of *E. coli* strains unable to ferment sucrose and glucose in the presence or absence of *P. aeruginosa* in the culture media. (a,b): CFUs of *E. coli Δpgi* core glycolysis mutant (a) or *E. coli Δldha* lactate dehydrogenase mutant (b) at 24 hours in single LB cultures or co-cultured with PA14 [n=6]. Plain LB and LB supplemented with 4% glucose or sucrose was used. **=p<0.005. Error bars represent standard deviation of the mean.

a

µg/ml	Sucrose		D-Glucose		Fructose	
**Diet**	PA14 + E.coli	PA14	PA14 + E.coli	PA14	PA14 + E. coli	PA14
Protein	3.5	0	1.5	2.3	0	0
Fat	0	0	0	0	0	1.6
Carbs	0	**46.0**	**17.3**	**21.1**	1.4	0.3
Control	0	13.0	5.5	0.7	1.7	0

b

µg/ml		D-lactic	SEM	L-lactic	SEM	Acetic	SEM
Protein	PA14 + E. coli	0		4,85	±3,41	96,72	±33,32
	PA14	0		23,92	±15,41	43,54	±43,54
Fat	PA14 + E. coli	**10,14**	±10,14	**31,78**	±12,39	35,55	±9,86
	PA14	0		21,28	±4,52	93,36	±21,99
Carbs	PA14 + E. coli	1,33	±1,33	7,81	±4,10	72,90	±25,21
	PA14	0		14,28	±5,22	50,54	±14,03

Suppl. Figure 6: Concentration of sugars and acetic and lactic acid in the feces of mice reared on nutrient-defined diets. (a,b): Sucrose, D-glucose and Fructose (a) and lactic and acetic acid (b) concentration (µg/ml) in fecal samples of mice reared on a fat-, protein, carbohydrate- based on control chaw diet [n=6]. Data are presented as mean ± standard error of mean.
